# Knockdown of HIF-1α by siRNA-expressing plasmid delivered by attenuated *Salmonella* enhances the antitumor effects of cisplatin on prostate cancer

**DOI:** 10.1038/s41598-017-07973-4

**Published:** 2017-08-08

**Authors:** Junlian Gu, Yang Li, Jun Zeng, Bo Wang, Kun Ji, Yufeng Tang, Qing Sun

**Affiliations:** 1 Department of Pathology, Qianfoshan Hospital Affiliated to Shandong University, Jinan, 250014 China; 2 Department of Pathophysiology, Prostate Diseases Prevention and Treatment Research Center, Norman Bethune College of Medicine, Jilin University, Changchun, 130021 China; 3 Department of Medical Genetics and Cell Biology, Guangzhou Medical University, Guangzhou, 510170 China; 4 Department of Pathology, The Second Clinical Medical School of Inner Mongolia University for the Nationalities (Inner Mongolia General Forestry Hospital), Yakeshi, 022150 Inner Mongolia China; 5 Department of Pathophysiology, Shenyang Medical College, Shenyang, 110034 Liaoning China; 6 Department of Orthopedic Trauma, Qianfoshan Hospital Affiliated to Shandong University, Jinan, 250014 China

## Abstract

Resistance to cisplatin (DDP) and dose-related toxicity remain two important obstacles in the treatment of prostate cancer (PCa) patients with DDP-based chemotherapy. We have investigated whether the knockdown of hypoxia-inducible factor-1 alpha (HIF-1α) by siRNA could enhance the antitumor activity of DDP, and aimed to determine the underlying mechanisms. Intravenous injection of attenuated *Salmonella* carrying a HIF-1α siRNA-expressing plasmid was used to knockdown HIF-1α in a PC-3 xenograft model. The *in vitro* and *in vivo* effects of HIF-1α siRNA treatment and/or DPP on PCa cell proliferation, apoptosis, glycolysis, and production of reactive oxygen species (ROS) were assessed by examining molecular markers specific to each process. The results demonstrated that the treatment of tumor-bearing mice with attenuated *Salmonella* carrying the HIF-1α siRNA plasmid greatly enhanced the antitumor effects of low-dose DDP. Further mechanistic studies demonstrated that knockdown of HIF-1α improved the response of PCa cells to DDP by redirecting aerobic glycolysis toward mitochondrial oxidative phosphorylation, leading to cell death through overproduction of ROS. Our findings indicate that DDP-based chemotherapy combined with targeting the HIF-1α-regulated cancer metabolism pathway might be an ideal strategy to treat PCa.

## Introduction

Prostate cancer (PCa) has become the most common cancer in men, accounting for 26% of all cancers, and 9% of cancer-related deaths in males^1^. Cisplatin (DDP) is an effective chemotherapeutic drug for many cancers^2^. However, DDP therapy is not recommended for PCa patients due to drug resistance^3, 4^. Although DDP resistance can be overcome by elevating the dosage, high doses of DDP often cause severe side effects such as ototoxicity, nephrotoxicity, peripheral neuropathy, gastrointestinal dysfunction, and myelosuppression. These adverse events usually lead to drug discontinuation and limited therapeutic efficacy^5^. One promising strategy is to pharmacologically or genetically (through gene therapy) sensitize cancer cells, enabling low-dose DDP to achieve a therapeutic effect, while avoiding the severe side effects of high-dose DDP.

Unlike normal tissue, PCa cells maintain high aerobic glycolytic rates and thus produce abundant lactate and pyruvate. This phenomenon has historically been known as the Warburg effect^6^. Importantly, cancer cells preferentially use the glycolysis pathway even in the presence of ample oxygen. The preferential reliance of cancers on glycolysis correlates with recurrence, progression, metastasis, and poor clinical outcomes in PCa patients^7^. Additionally, the activities of enzymes in the glycolysis pathway are consistently elevated in PCa cells^8–12^. Hypoxia-inducible factor-1 alpha (HIF-1α) is a critical transcription factor that activates the expression of nearly all enzymes involved in glycolysis. It is well established that HIF-1α is upregulated and promotes tumor metastasis in malignant tumors^13^. The inhibition of HIF-1α may alter the preferential metabolic pathway in cancer cells from glycolysis to oxidative phosphorylation to inhibit tumor metastasis^14^. When HIF-1α is downregulated in ovarian cancer cells, the cells become sensitive to chemotherapy through the downregulation of glycolytic enzyme activity both *in vitro* and *in vivo* ^15^. Therefore, utilization of small interfering (si)RNA technology to silence HIF-1α activity in PCa may be an effective gene therapy to overcome drug resistance through the regulation of glycolysis.

A major challenge for gene therapy is to develop an efficient gene delivery system that can selectively target tumors. Genetically attenuated bacteria such as attenuated *Salmonella* offers promise as an anticancer vector and has been widely used as a tool to deliver plasmids that express siRNA *in vivo* ^16–21^. Our previous studies have demonstrated that a combination of low-dose DDP with gene therapy expressing tumor protein p53 (p53) and mouse double minute 2 homolog (MDM2) siRNA, delivered by attenuated *Salmonella enterica serovar Typhi vaccine strain Ty21a* (*Salmonella Typhi Ty21a*), synergistically inhibited ovarian cancer and PCa growth without damaging normal tissues^22–25^. Here, we tested our hypothesis that knockdown of HIF-1α by siRNA gene therapy delivered by the attenuated *Salmonella Typhi Ty21a* is a promising strategy to increase the sensitivity of PCa to DDP from the perspective of targeting cancer-specific metabolism. Our results showed that DDP combined with the attenuated *Salmonella* carrying the HIF-1α-siRNA plasmid had an optimally therapeutic effect on PCa when compared to DDP alone in a nude mouse xenograft model. Mechanistic studies demonstrated that the combination therapy could effectively induce apoptosis of cancer cells by inhibiting glycolysis metabolism. Importantly, few toxic side effects associated with the combination therapy were observed.

## Results

### HIF-1α was upregulated in PCa cell lines and primary human PCa cells

**Figure 1:**
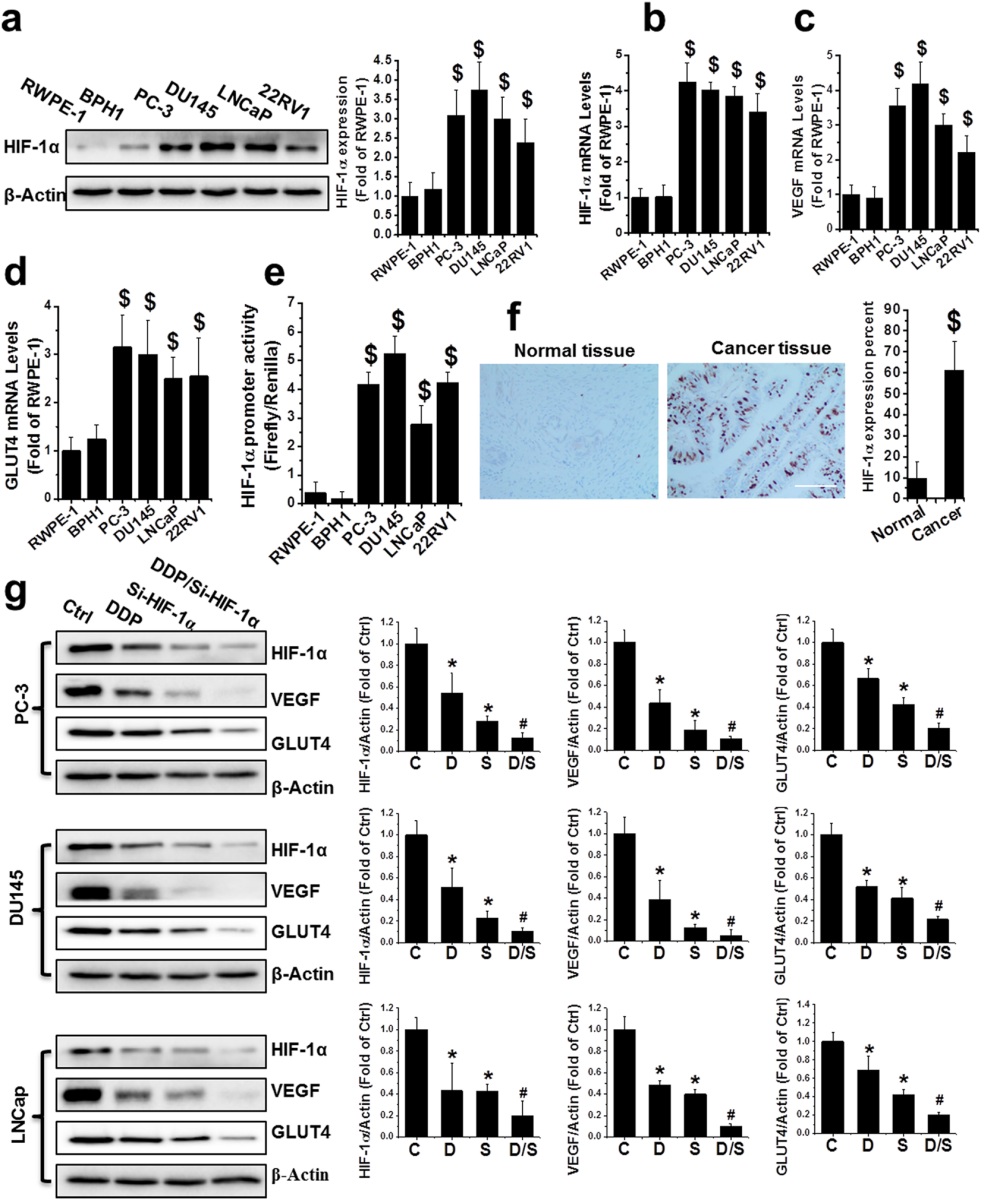
Upregulation of HIF-1α in human PCa. (**a**) HIF-1α protein was detected by western blot in nonmalignant (RWPE-1 and BPH1) and PCa cell lines (PC-3, DU145, LNCaP, and 22RV1) as indicated. (**b**–**d**) Total RNA extracted from RWPE-1, BPH1, PC-3, DU145, LNCaP, and 22RV1 cells was subjected to qRT-PCR for HIF-1α (**b**), VEGF (**c**) and GLUT4 (**d**). (**e**) The HIF-1α promoter-driven reporter (firefly luciferase) and a control vector (Renilla luciferase) were co-transfected into RWPE-1, BPH1, PC-3, DU145, LNCaP, and 22RV1 cells for measurement of luciferase activity. HIF-1α promoter activity was calculated as a ratio of firefly to Renilla activity. (**f**) Human normal and malignant tissue (Gleason score 9) sections were probed with HIF-1α antibody (scale bars, 100 µm). (**g**) Protein expression of HIF-1α, VEGF, and GLUT4 were examined with western blot, in PC-3, DU145, and LNCaP cells after various treatments as indicated. Data are expressed as mean ± SD of seven independent experiments. ^$^p < 0.05 versus RWPE-1 or BPH1 cells or normal tissue. *p < 0.05 versus control group. ^#^p < 0.05 versus si-HIF-1α or DDP group. Original blots are shown in Supplementary Figure 5. C: Ctrl; D: DDP; S: si-HIF-1α; D/S: DDP/si-HIF-1α.

Western blot analyses were performed to compare HIF-1α protein expression in four representative PCa cell lines (androgen-receptor-negative PC-3 and DU145, androgen-responsive LNCaP, and castration-resistant 22RV1) and in two non-malignant prostatic epithelial cell lines (RWPE-1 and BPH1). HIF-1α protein levels were markedly elevated in malignant cell lines compared to benign cell lines (Fig. 1a). Consistently, HIF-1α mRNA (Fig. 1b) was also upregulated in the PCa cell lines. Moreover, expression of vascular endothelial growth factor (VEGF) and glucose transporter type 4 (GLUT4), which are regulated by HIF-1α, were significantly increased as determined by quantitative real-time PCR (qRT-PCR, Fig. 1c,d). Furthermore, HIF-1α transcriptional activity, measured using a reporter gene assay, was upregulated in the malignant cells compared to the benign cells (Fig. 1e). Moreover, immunohistochemical (IHC) analysis showed a significantly higher percentage of HIF-1α-positive cells in primary PCa tissue (61.26%) compared to normal tissue (9.44%), and HIF-1α expression was primarily localized in the nucleus (Fig. 1f).

Next, we aimed to determine whether treatment with DDP and/or the si-HIF-1α plasmid could efficiently downregulate HIF-1α expression. Three PCa cell lines, PC-3, DU145, and LNCaP, were used to test the knockdown efficiency of HIF-1α expression following treatment with si-HIF-1α plasmid and/or DDP. The si-HIF-1α plasmid transfection alone significantly reduced HIF-1α protein expression in all three cell lines compared to the vehicle group (Fig. 1g), suggesting effective silencing of HIF-1α expression by the siRNA-expressing plasmid. DDP alone also slightly inhibited HIF-1α expression in PC-3, DU145, and LNCaP cells (Fig. 1g). The combination of DDP and the si-HIF-1α plasmid inhibited HIF-1α protein expression even further, reaching inhibition of 87.4%, 89.6%, and 80.7% in PC-3, DU145, and LNCaP cells, respectively. Consistently, VEGF and GLUT4, which are both downstream of HIF-α, were also downregulated by DPP and si-HIF-1α alone, and the greatest reduction occurred when the cells were treated in combination with DDP and si-HIF-1α (Fig. 1g).

### Combination of si-HIF-1α and DDP therapy markedly reduced cell viability, proliferation, and colony formation capability in PCa cell lines

**Figure 2:**
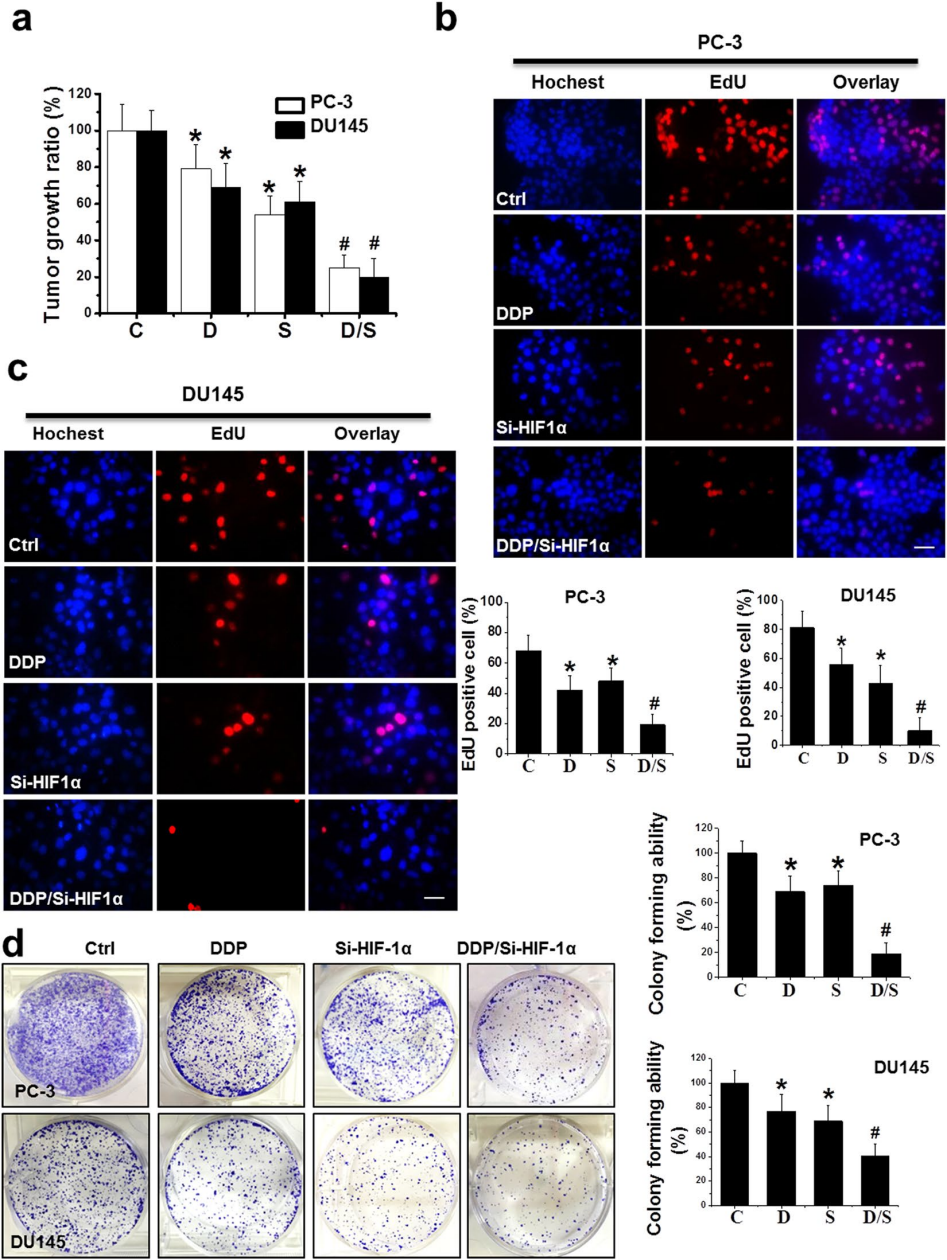
Inhibition of PCa cell growth *in vitro* by various treatments. (**a**) Cell viability was assessed using a MTT assay in PC-3 and DU145 cells after various treatments as indicated. (**b**,**c**) Cell proliferation was examined using an EdU assay in PC-3 (**b**) and DU145 (**c**) cells after various treatments as indicated (scale bars = 25 µm). (**d**) Colonies of PC-3 and DU145 cells were stained with crystal violet 14 days after treatment and counted. Data were presented as mean ± SD of three independent experiments. *p < 0.05 versus control group; ^#^p < 0.05 versus si-HIF-1α or DDP group.

MTT (Fig. 2a) and EdU (Fig. 2b,c) assays were both used to evaluate the effects of combination treatment on cell viability and cell proliferation in PC-3 and DU145 cells. The results showed that treatment with DDP, si-HIF-1α, and DDP/si-HIF-1α resulted in a significant reduction of cell viability and cell proliferation in PC-3 and DU145 cells 48 h after treatment, with a maximal reduction obtained by the DDP/si-HIF-1α treatment (Fig. 2a–c).

Colony assays reflected the capability of single cells to survive and proliferate under various treatment conditions. DDP or si-HIF-1α alone moderately but significantly inhibited colony formation, while the combination treatment dramatically decreased the colony formation capability of the cells (Fig. 2d).

### Combination treatment showed remarkable antitumor activity in a PCa xenograft model

**Figure 3:**
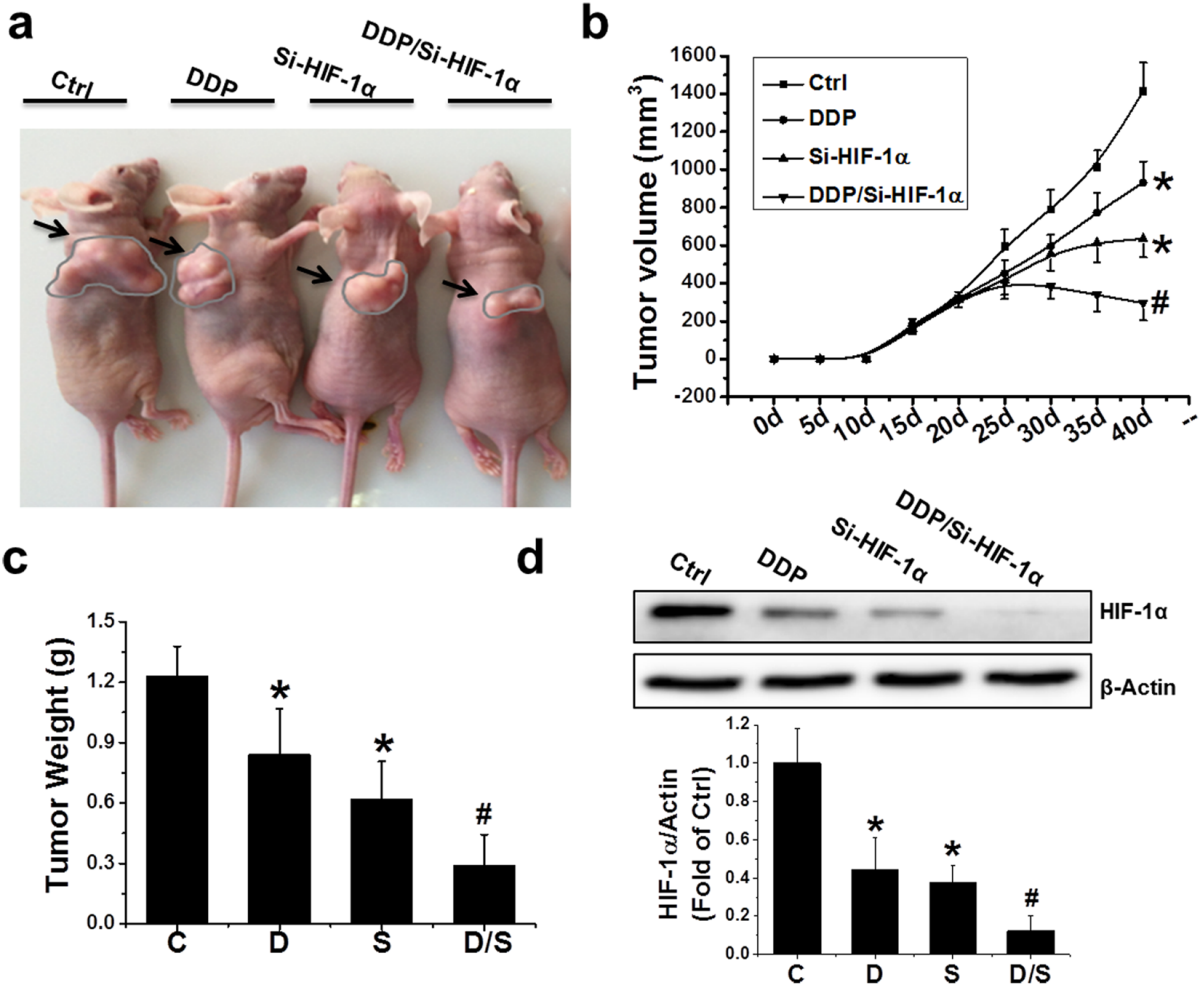
Inhibition of tumor growth *in vivo* by various treatments. (**a**) Macroscopic view of mouse tumors at the end of the study. (**b**) Tumor growth curves from day 0 to 40, with tumor sizes measured every 5 days, in various treatment groups as indicated. (**c**) Wet weight of tumors measured at the end of the study. (**d**) Protein expression of HIF-1α examined by western blot in PC-3 xenografts exposed to various treatments. Data were presented as mean ± SD (n = 6). *p < 0.05 versus control group; ^#^p < 0.05 versus si-HIF-1α or DDP group. The original blots are presented in Supplementary Figure 6.

PCa xenografts were established through the subcutaneous injection of PC-3 cells on the right flank of male nude mice. Two weeks after the cell injection, tumor-bearing mice received treatments with DDP, si-HIF-1α, or DDP/si-HIF-1α. The representative tumor xenograft for each treatment is shown in Fig. 3a. All treatments caused a significant inhibition of tumor expansion compared to the control, but the combination treatment with DDP and si-HIF-1α had the greatest antitumor effect. At sacrifice, the average tumor size of the control, DDP, si-HIF-1α, and combination groups were 1413 mm^3^, 932 mm^3^, 636 mm^3^, and 296 mm^3^, respectively (Fig. 3b). Consistently, the average tumor weight in the DDP/si-HIF-1α group was significantly lower than those in the other groups (Fig. 3c). The combination treatment also led to a significantly greater decrease of HIF-1α protein in the PCa xenograft compared to treatment with DDP or si-HIF-1α alone (Fig. 3d).

### Combination treatment with DDP and si-HIF-1α caused tumor cell apoptosis, but not cell cycle arrest in PCa xenografts

**Figure 4:**
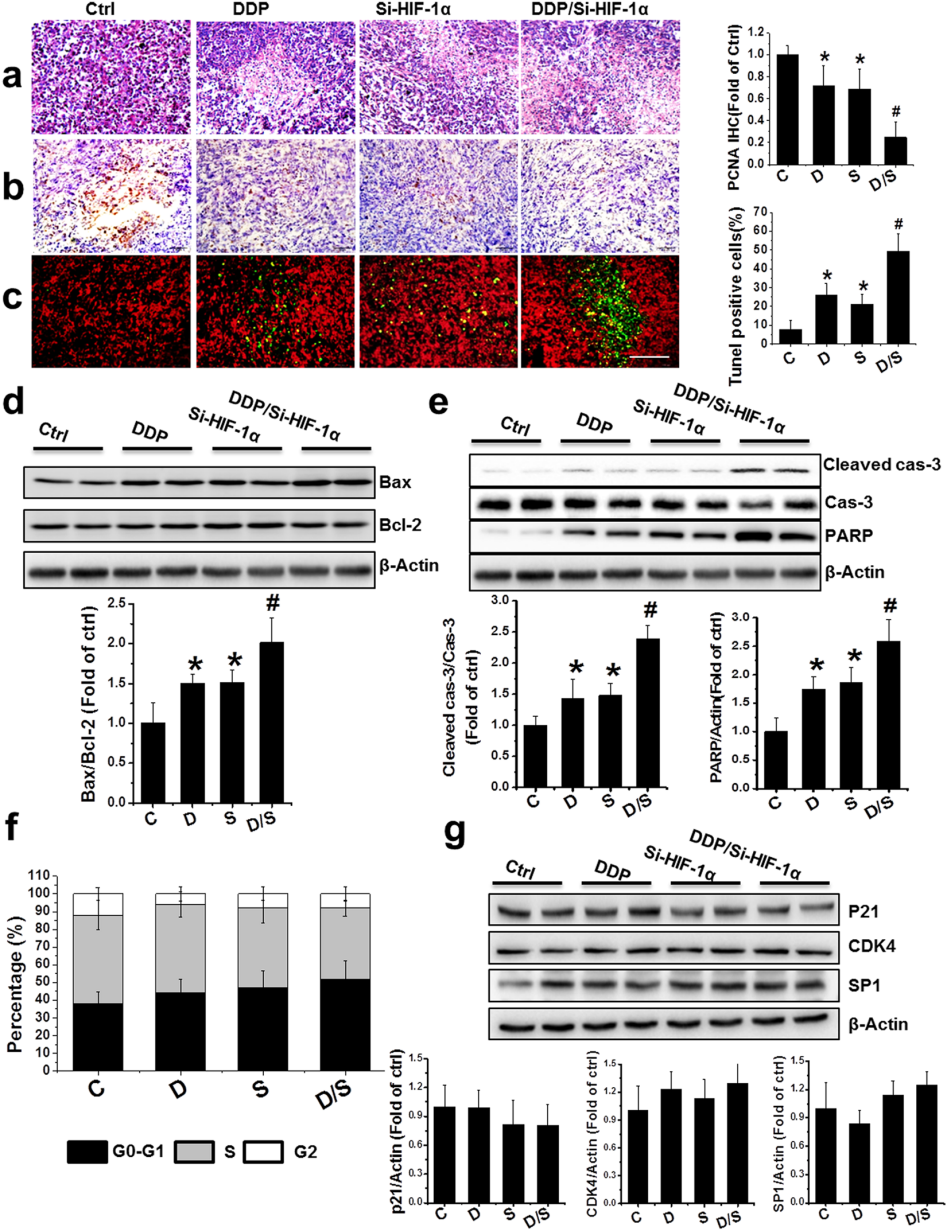
Effect of treatment with DDP and/or HIF-1α siRNA on the induction of apoptosis in PCa xenografts. (**a**–**c**) Representative images of H&E (**a**), PCNA (**b**), and TUNEL (**c**) in the xenografts with treatments as indicated (scale bars, 100 µm). (**d**,**e**) Bax/Bcl-2 ratio (**d**) and protein abundance of cleaved forms of caspase-3 and PARP (**e**) were examined in PCa xenografts by western blot. (**f**) Values represent the percentage of cells in each phase of the cell cycle that were detected by flow cytometry analysis. (**g**) Western blot for protein expression of p21, CDK4, and SP1 after various treatments. Data were presented as mean ± SD (n = 6). *p < 0.05 versus control group; ^#^p < 0.05 versus si-HIF-1α or DDP group. The original blots are presented in Supplementary Figure 7.

When examined by hematoxylin and eosin (H&E) staining, a number of necrotic foci and tissue disorganization were observed in the DDP, si-HIF-1α, and DDP/si-HIF-1α groups, with the combination group demonstrating the most serious injury (Fig. 4a). The expression of proliferation cell nuclear antigen (PCNA), which is a marker for proliferation, was significantly reduced in groups treated with combination therapy, compared to other treatments (Fig. 4b). The number of cells that stained positive for TUNEL, a marker of DNA fragmentation and apoptosis, increased significantly in the DDP, si-HIF-1α, and DDP/si-HIF-1α groups compared to control (Fig. 4c). The DDP/si-HIF-1α combination treatment caused the highest level of apoptosis. Consistent with this, Western blot analysis revealed an increase in markers of apoptosis in the DDP/si-HIF-1α group compared to the other groups, including an increase in the Bax/Bcl-2 ratio and the induction of caspase-3 and PARP cleavage (Fig. 4d,e). Differences in apoptosis and related protein changes were further confirmed by an *in vitro* study using PC-3 cells, which showed that DNA fragmentation, as well as the Bax/Bcl-2 ratio and induction of caspase-3 and PARP cleavage, were significantly increased in the DDP/si-HIF-1α group compared to the other groups (Supplementary Figure 2a,b).

Another possible reason for the regression of the PC-3 xenografts with DDP/si-HIF-1α therapy may be due to cell cycle arrest. Therefore, the cell cycle distribution of xenografts was measured using flow cytometry. However, there was no significant change in the percentage of G1-phase cells in the xenograft treated with DDP alone, si-HIF-1α alone, or in combination (Fig. 4f). Consistently, the expression of cell cycle regulators such as p21, cyclin-dependent kinase 4 (CDK4), and specificity protein 1 (SP1) also had no significant change among all groups, as measured by Western blot analysis. These data suggest no significant change in cells arrested in the G1-phase (Fig. 4g).

### DDP and/or HIF-1α siRNA induced apoptosis mediated by reactive oxygen species overproduction

**Figure 5:**
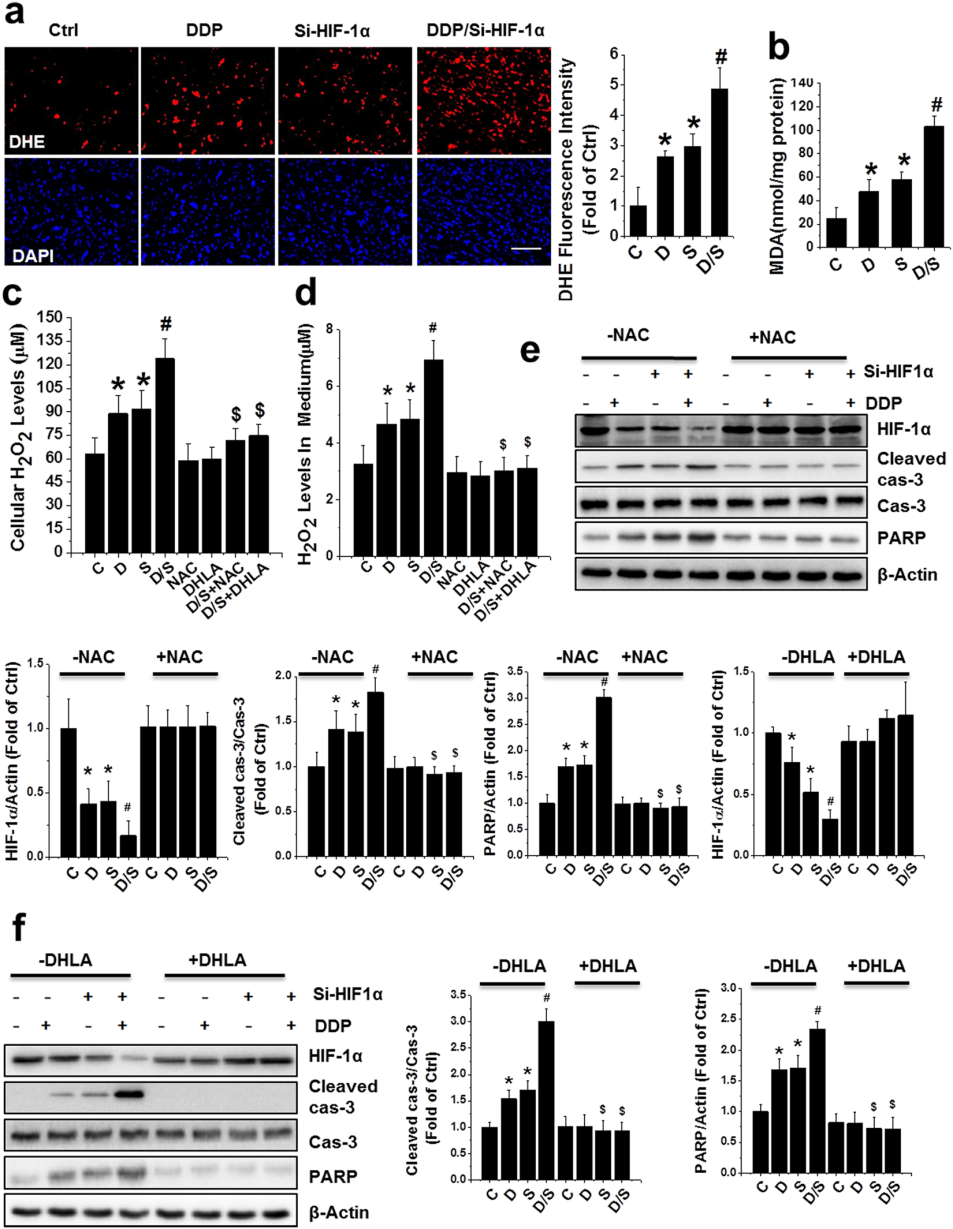
ROS overproduction mediated apoptosis induced by combined treatment of DDP and HIF-1α in PC-3 xenografts and cell culture. (**a**) ROS monitored by DHE (red) and nuclei by DAPI (blue) staining in PCa xenografts (scale bars, 50 µm). (**b**) MDA formation of PCa xenografts was examined after various treatments (**c**,**d**). PC-3 cells were treated with DDP, si-HIF-1α plasmid, or both, in the presence or absence of NAC (5 mM) or DHLA (0.25 mM) for 24 h. Total lysates (**c**) and culture media (**d**) were used to detect cellular H_2_O_2_ level. (**e**,**f**) Western analysis for HIF-1α as well as cleaved caspase-3 and PARP in PC-3 cells following various treatments. Data were presented as mean ± SD of three independent experiments. *p < 0.05 versus control group; ^#^p < 0.05 versus si-HIF-1α or DDP group; ^$^p < 0.05 versus DDP/si-HIF-1α group. The original blots are presented in Supplementary Figure 8.

Next, we tested whether HIF-1α downregulation sensitized PC-3 cells to apoptosis through overproduction of ROS. We examined the effect of the combined treatment with DDP and si-HIF-1α on levels of ROS with a dihydroethidium (DHE) assay. The combination treatment significantly enhanced DHE fluorescence intensity in PCa xenografts and PC-3 cells (Fig. 5a and Supplementary Figure 3a, respectively). Further, the levels of cellular malondialdehyde (MDA) were higher in PCa xenografts and PC-3 cells following combination treatment (Fig. 5b and Supplementary Figure 3b). Moreover, the H_2_O_2_ level was significantly increased in both the cytoplasm and culture medium of PC-3 cells after the combined treatment of DDP with si-HIF-1α (Fig. 5c,d). However, ROS production induced by combination treatment was markedly abolished by ROS scavengers, including N-acetyl cysteine (NAC) and dihydrolipoic acid (DHLA; Fig. 5c,d and Supplementary Figure 3a,b). Consistently, caspase-3 and PARP cleavage induced by the combination treatment was remarkably decreased when treated with NAC (Fig. 5e) or DHLA (Fig. 5f). These data clearly suggest that HIF-1α knockdown sensitizes PCa xenografts and cell lines to DDP-induced apoptosis by overproduction of ROS.

### Overexpression of LDH-A partially blocked apoptosis induced by combined treatment with DDP and HIF-1α siRNA

**Figure 6:**
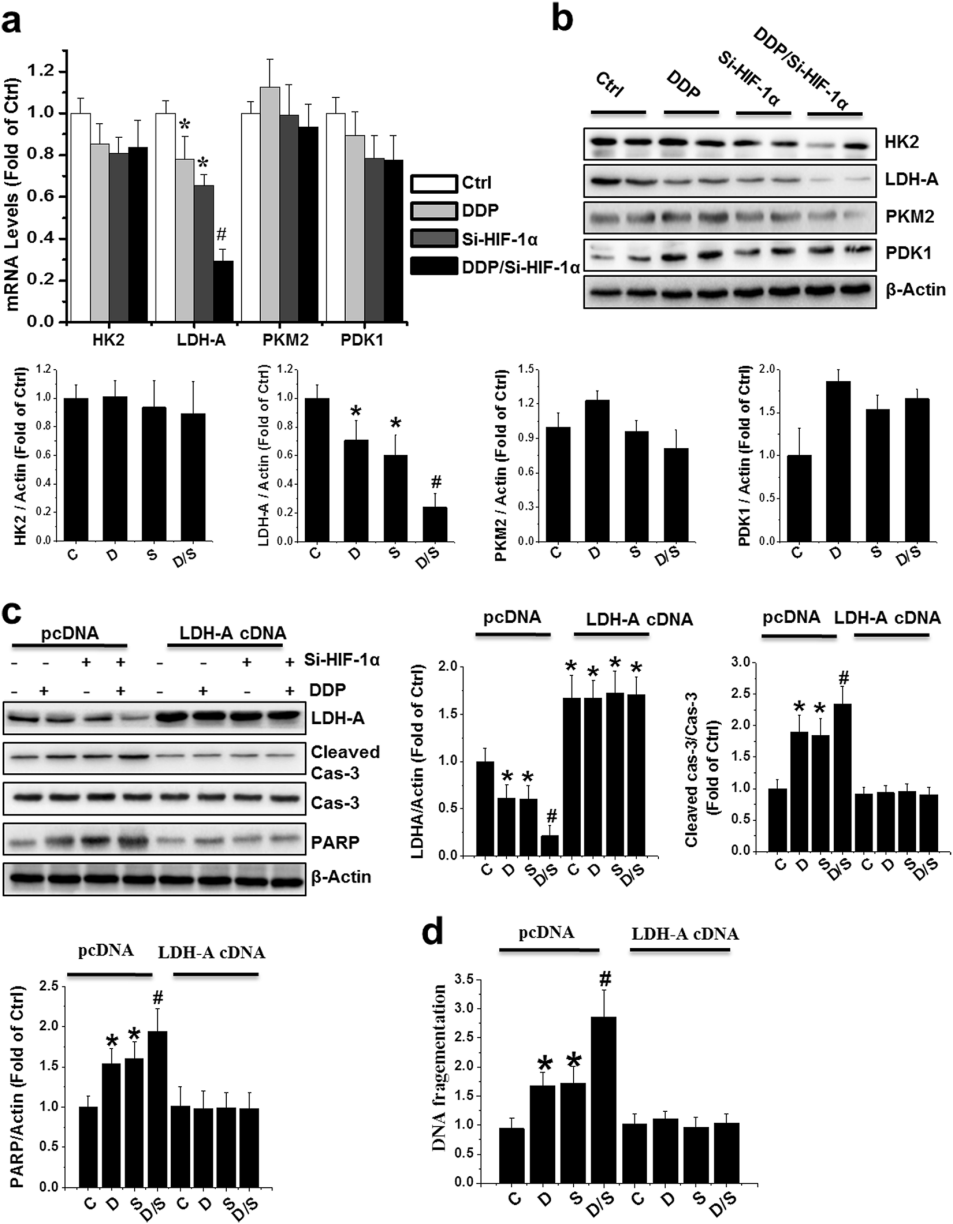
LDH-A overexpression blocked apoptosis induced by DDP and/or HIF-1α siRNA. (**a**,**b**) mRNA and protein expression of HK2, LDH-A, PKM2 and PDK1 was examined with qRT-PCR (**a**) and western blot (**b**) in PCa xenografts. (**c**,**d**) PC-3 cells were transfected with a human LDH-A cDNA construct or empty vector (pcDNA) for 48 h. Following 24 h of cDNA treatment, the cells were treated by transient transfection with si-HIF-1α alone, DDP alone, or both in combination. Cell lysates were subjected to western blot analysis with the indicated antibodies (**c**) and an apoptosis ELISA (**d**). Data were presented as mean ± SD of three independent experiments. ^*^p < 0.05 versus control group; ^#^p < 0.05 versus si-HIF-1α or DDP group. The original blots are presented in Supplementary Figure 9.

Glycolysis-related enzymes, such as hexokinase 2 (HK2), pyruvate kinase isozyme M2 (PKM2), phosphoinositide-dependent kinase 1 (PDK1), and lactate dehydrogenase A (LDH-A), are known to be regulated by HIF-1α. As expected, we found that LDH-A was significantly reduced at both the mRNA and protein levels following combination treatment in PCa xenografts (Fig. 6a,b), as well as in PC-3 cells *in vitro* (Supplementary Figure 4a,b). Importantly, glucose uptake levels and lactate production are highly dependent on the rate of glycolysis. Decreased lactate production and glucose uptake were observed in PC-3 cells when treated with DDP/Si-HIF-1α, compared to other treatments (Supplementary Figure 4c,d).

LDH-A drives the switch of the metabolic pathway from mitochondrial oxidative phosphorylation to glycolysis. Thus, we tested whether overexpression of LDH-A could reduce DDP-triggered apoptosis. LDH-A overexpression reduced PARP and caspase-3 cleavage, as determined by Western blot (Fig. 6c), and reduced histone-associated DNA fragmentation, as determined by ELISA (Fig. 6d), following combination treatment in PC-3 cells. The above data strongly suggests that HIF-1α-silencing enhances the sensitivity of PCa cells to DDP through a change in the preferential metabolic pathway from glycolysis to oxidative phosphorylation, leading to ROS production and subsequent induction of apoptosis.

### DDP/si-HIF-1α treatment caused little systemic toxicity in mice

**Figure 7:**
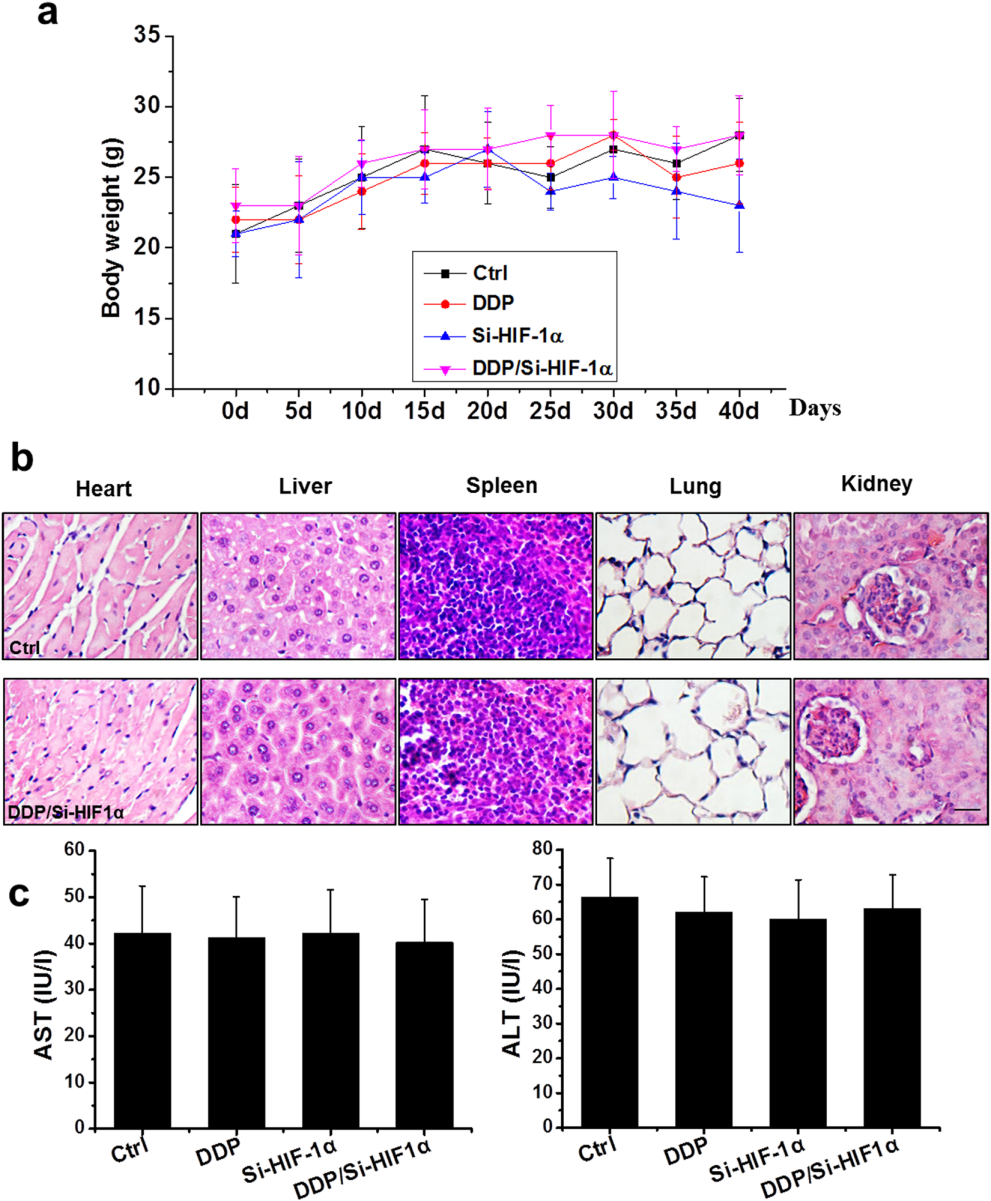
Systemic toxicity caused by HIF-1α siRNA and/or DDP treatment in mice. (**a**) Mean body weights. (**b**) H&E staining of the heart, liver, spleen, lung, and kidney, 40 days following various treatments as indicated (scale bars = 25 µm). (**c**) Effects of various treatments on activity of serum ALT and AST. Data were presented as mean ± SD (n = 6).

We assessed systemic toxicity in mice caused by the treatment of DDP alone, si-HIF-1α alone, and in combination. There was no difference in body weight among the groups (Fig. 7a). Histopathological examination by H&E staining showed that DDP/si-HIF-1α treatment caused only minor toxicity to the tissues of the major internal organs (Fig. 7b). There was no difference in hepatic function caused by combination therapy, as measured by serum levels of aspartate aminotransferase (AST) and alanine aminotransferase (ALT; Fig. 7c). Moreover, no abnormalities such as appetite loss, fur ruffling, or behavioral changes were noticed following the combination treatment (data not shown).

## Discussion

We tested our hypothesis that the sensitivity of PCa cells to DDP could be enhanced by reducing HIF-1α-regulated cancer metabolism. First, we demonstrated that downregulating HIF-1α through RNA interference could sensitize PCa xenografts and cell lines to DDP-induced apoptosis. Next, we investigated the underlying mechanisms of the enhanced sensitivity of cancer cells to DDP by HIF-1α downregulation, and found a switch of the metabolic pathway from glycolysis to oxidative phosphorylation, which caused an increase in ROS and sensitized the cells to DDP.

To overcome the resistance of the tumor to DDP and dose-related toxicity, efforts have been made to develop a combination strategy to improve the therapeutic effects of DDP. Recently, a combination of DDP and gene therapy aimed to improve antitumor efficacy has become appreciated^22–24, 26, 27^. For instance, it has been shown that when combined with adenovirus-mediated phosphatase and tensin homolog (PTEN), DDP has powerful antitumor activity in human small-cell lung cancer^28^. Also, NK4 gene therapy combined with low-dose DDP has been reported to be an effective regimen for oral squamous cell carcinoma^29^. Our previous study also demonstrated that DDP combined with gene therapy for both p53 and MDM2-siRNA greatly suppressed PCa and ovarian tumor growth *in vitro* and *in vivo* ^22, 23^.

It has been well established that HIF-1α is upregulated in primary PCa and is important for invasion and migration in PCa^30–32^. Some patients with advanced PCa have high nuclear HIF-1α expression, which correlates with metastases at relapse, as well as reduced disease-specific survival^33^. We confirmed that HIF-1α was remarkably upregulated in human prostate tumors and PCa cell lines, but not in corresponding normal tissues or nonmalignant cells. Previous studies have also reported that HIF-1α is involved in promoting resistance of tumors to chemo- and radiotherapy^30^. This suggests that HIF-1α status might be a valid marker for predicting the sensitivity of cancers to chemotherapies. It has also been reported that DDP could downregulate HIF-1α by promoting HIF-1α degradation through the proteasome degradation pathway^15^. In our model, we found that HIF-1α was significantly decreased following combined treatment with DDP and si-HIF-1α, and the subsequent transcriptional activation of HIF-1α target genes, such as VEGF and GLUT4, was blocked. In this study, we also demonstrated for the first time that *in vivo* transfection of PC-3 xenografts with an HIF-1α-siRNA plasmid delivered by attenuated *Salmonella Typhi Ty21a* significantly improved the antitumor effects of DDP.

Next, we further explored the mechanism of the enhanced PCa sensitivity to DDP by downregulating HIF-1α in cell lines and xenografts. One possible mechanism of drug resistance is through the inactivation of apoptosis. Most anticancer therapies, including chemotherapy and radiotherapy, exert their efficacy by activating apoptosis signaling pathways in cancer cells. This study revealed that downregulation of HIF-1α using siRNA combined with DDP markedly increased apoptosis, as shown by multiple apoptosis markers such as cleaved caspase-3 and PARP. Another possible reason for the regression of PC-3 xenografts with DDP/si-HIF-1α therapy might be due to cell cycle arrest. To our surprise, combination therapy affects neither cell cycle distribution nor the expression of cell cycle regulators such as p21 and CDK4 in PC-3 xenografts. It has been proposed that HIF-1α is a major regulator of cell cycle arrest under hypoxic conditions^34^. Knockdown of HIF-1α can result in cell cycle disruption and alters the cellular response to chemotherapeutic drugs in a SP1-dependent manner^35^. Therefore, we analyzed the expression of SP1 and found no significant difference among all groups in our model. Taken together, these data suggested that the principal cause of enhanced tumor regression in the PC-3 xenografts following combination therapy depended on the induction of apoptosis, not the promotion of cell cycle arrest. However, the latter might still play a major role of tumor inhibition in other models^35, 36^.

Most chemotherapeutic agents, such as paclitaxel, doxorubicin, and cisplatin/DDP, could elicit mitochondrial permeabilization and intrinsic apoptosis by perturbing glycolysis. Increasing evidence has demonstrated a possible link between glycolysis and resistance to apoptosis in tumor maintenance and progression^37^. For example, HIF-1α engages the glycolysis pathway by inducing the expression of many glycolytic enzymes such as HK1 and LDH-A to inhibit apoptosis^15, 38^. Additionally, downregulation of HIF-1α improves the response of treatment-resistant cells and induces apoptosis through ROS production. It has been reported that oxidative conditions or increased levels of ROS downregulates HIF-1α expression, and decreased ROS levels upregulates HIF-1α expression^39, 40^. NAC or DHLA, as scavengers of ROS, have also been reported to stimulate HIF-1α expression^39, 41–43^, which is consistent with our findings in this study. A recent study showed that reduction of LDH-A, a direct target gene of HIF-1α, decreased cellular transformation and significantly increased apoptosis, as well as delayed tumor development and metastases, indicating that LDH-A is involved in tumor initiation and progression^44^. Consistent with the above studies, we also found that the key glycolytic enzyme LDH-A was significantly reduced following combined treatment with si-HIF-1α and DDP. Further mechanistic studies found that overexpression of LDH-A largely blocked apoptosis that was induced by the combination treatment. Taken together, by reducing the glycolytic enzyme LDH-A, combined treatment of si-HIF-1α and DDP induces apoptosis in PCa cells, leading to increased ROS production, and subsequently inhibiting tumor growth.

In summary, we showed that silencing HIF-1α greatly enhanced the therapeutic efficacy of low-dose DDP on PCa in a xenograft model. We further demonstrated that downregulation of HIF-1α overcame chemotherapy resistance in PCa by redirecting the metabolic pathway from aerobic glycolysis to mitochondrial oxidative phosphorylation. This work provides novel insights into the role of HIF-1α in drug resistance, and our results may help to develop new effective approaches to the treatment of patients with advanced PCa.

## Methods and Materials

### Ethics Statement

This study was carried out strictly following the recommendations in the Guide for the Care and Use of Laboratory Animals, Eighth Edition (Library of Congress Control Number: 2010940400, revised 2011). All experimental procedures for animal studies were approved by the Institutional Animal Care and Use Committee of the Shandong University affiliated Qianfoshan hospital (protocol number: S030). All appropriate means were undertaken to minimize suffering.

### Normal and cancerous human prostate tissues

Both normal and malignant prostate tissue (Gleason score 9) were kindly provided by Inner Mongolia General Forestry Hospital (Yakeshi, China). Five samples of normal prostate tissue from benign areas of the prostatectomy specimens, and six samples of cancer tissues from radical prostatectomy specimens of patients with organ-confined tumors without previous therapy were used. Patients did not have other systemic diseases. The studies involving human specimens were approved by the Medical Ethics Committee of Inner Mongolia General Forestry Hospital Affiliated to Inner Mongolia University. Informed consent was obtained from each patient under an institutionally approved protocol. All methods were performed in accordance with the relevant guidelines and regulations of Inner Mongolia General Forestry Hospital Affiliated to Inner Mongolia University.

### Cell culture and treatment

The PCa cell lines PC-3, DU145, LNCaP, 22RV1, RWPE-1, and BPH1 were obtained from the Cell Bank of the Chinese Academy of Sciences (Shanghai, China) where they were characterized by mycoplasma detection, DNA-fingerprinting, isozyme detection, and cell vitality detection. They were cultured in Iscove’s modified Dulbecco’s medium (IMDM, Hyclone, Logan, UT, USA) supplemented with 10% (v/v) fetal bovine serum (FBS). The cells were treated with DDP (Sigma-Aldrich, St. Louis, MO) at 6 µg/mL for 48 h, as previously reported^23^. The transfection reagent used was Lipofectamine 2000 (Invitrogen, CA, USA). Approximately 6 × 10^3^ cells per well were transfected with the plasmids with or without DDP for 48 h, then cell viability was determined by (3-(4,5-dimethylthiazolyl-2)-2,5-diphenyltetrazolium bromide (MTT, Sigma-Aldrich) assay following standard protocols as previously described^25^.

### Construction of the si-HIF-1α plasmid and transfection of attenuated Salmonella Typhi Ty21a

The construction of the si-HIF-1α plasmid is shown in Supplementary Figure 1 and the attenuated *Salmonella Typhi Ty21a* was generously provided by Dr. Hohmann^45^ and stored in the Prostate Diseases Prevention and Treatment Research Center of Jilin University. The plasmid was transfected into competent cells by electroporation to generate bacteria expressing HIF-1α siRNA.

### Xenograft model

Athymic male nude mice (nu/nu; 8 weeks old; the Institute of Zoology, Chinese Academy of Sciences, Beijing, China) were housed in standard microisolator conditions free of specific pathogens in accordance with the Animal Care and Use protocol approved by Shandong university. These mice were used to establish a xenograft model. The PC-3 suspension (2 × 10^6^ cells in 100 µL PBS per mouse) was subcutaneously injected into the right flank of the animals. The tumor sizes were measured from the first day until 40 days post-cell injection, using calipers and the formula: V (volume) = LW^2^ × 0.52, where “L” represents the greatest length and “W” represents the perpendicular width. After tumors reached approximately 150–200 mm^3^ (~2 weeks), the animals were randomized into four groups (n = 6/group): normal control group, with an intraperitoneal injection (i.p.) of normal saline; DDP group, with i.p. injection of DDP; si-HIF-1α group, with transfection of si-HIF-1α; and DDP/si-HIF-1α group, with combined treatment of si-HIF-1α and DDP. The bacteria, carrying the si-HIF-1α plasmid at 1 × 10^7^ CFU/100 µL, were injected through the tail vein at day 14 and day 28 post-PC-3 cell inoculation as previously described^18, 22^. After the first bacterial injection, DDP was administered (2.0 mg/kg, i.p.) twice a week for 2 weeks as previously described^23, 24^.

### Western blot assay

Western blotting was performed as previously described^22, 23, 46^. The lysates from the xenografts or cell lines with different treatments were homogenized in RIPA lysis buffer using a homogenizer. The supernatants were collected by centrifugation (12,000 rpm at 4 °C for 25 min; Beckman GS-6R). Protein was resolved by electrophoresis and transferred onto polyvinylidene fluoride (PVDF) membranes (Millipore, Bedford, MA, USA). The membranes were probed with monoclonal HIF-1α (NB100–105, Novus biological, Littleton, CO, USA), Bax (2772 S, Cell signaling, Boston, MA, USA), Bcl-2 (3498S, Cell signaling), caspase-3 (9661S, Cell signaling), PARP (5625, Cell signaling), and PCNA (18197, Abcam, Cambridge, MA, USA), as well as p21 (Sc817, Santa Cruz, CA, USA), CDK4 (Sc70831, Santa Cruz), SP1 (ab27595, Abcam), HK2 (2772S, Cell signaling), PKM2 (4053S, Cell signaling), LDH-A (3582S, Cell signaling), and PDK1 (3820 S, Cell signaling). Following incubation with the primary antibody overnight at 4 °C, membranes were washed with Tris-buffered saline (pH 7.2) containing 0.05% Tween-20 and subsequently incubated with horse radish peroxidase (HRP)-conjugated anti-mouse (Sc-2005, Santa Cruz) or anti-rabbit (Sc-2357, Santa Cruz) secondary antibody for 1 h at room temperature. Bands of interest were analyzed using the ChemiDocTM Touch Imaging System (BIO-RAD, Hercules, CA, USA).

### TUNEL and EdU labeling assays

Terminal deoxynucleotidyl transferase dUTP nick-end labeling (TUNEL) staining was performed as previously reported^23^. Briefly, the xenografts from each treatment were fixed in buffered neutral 10% formalin, dehydrated in a series of graded alcohol, embedded in paraffin, and sectioned (5 μm). Deparaffinized and rehydrated slides were used for TUNEL staining following the manufacturer’s instructions (Promega, Madison, WI, USA). Apoptosis of cells was quantitatively analyzed by counting TUNEL-positive cells from 10 randomly selected fields in three slides per mouse, from at least five mice in each group. 5-ethynyl-2′-deoxyuridine (EdU) was detected with Click-iT® EdU Alexa Fluor® 647 Imaging Kit (C10640, Invitrogen, Carlsbad, CA). Imaging was performed on a fluorescent microscope (Nikon, Melville, NY, USA) and the signals were quantified by ImageJ software.

### ROS measurement

PC-3 xenograft tissues were immediately frozen in optimal cutting temperature (OCT) compound and frozen sections (10 µm) were prepared at −20 °C using a cryostat (Leica, Electronic). The sections were thawed at room temperature for 30 min and then incubated in dihydrogen ethidium (10 μM; DHE, Sigma-Aldrich) for 30 min at 37 °C in the dark. Next, the sections were washed three times and incubated with DAPI (2 µg/mL). Digital images were captured by a fluorescent microscope (Nikon), and the fluorescence intensity, normalized to that of the control, was quantified using ImageJ software.

Following treatment, PC-3 cells (1.0 × 10^5^ cells/well) cultured on six-well plates were incubated with DHE dye (5 μm/mL) diluted in Hank’s balanced salt solution (HBSS) for 30 min at 37 °C in the dark. Images were captured on a fluorescence microscope and ROS signals were quantified using ImageJ software. The H_2_O_2_ concentrations were measured using an H_2_O_2_ assay kit (Beyotime, Beijing, China) according to the manufacturer’s instructions.

### Colony formation analysis

Colony formation assays were performed as previously described^22, 23^. Briefly, cells were seeded in six-well plates (500 cells/well) and cultured for 14 days. Colonies were fixed with 10% formaldehyde for 15 minutes, and stained with 0.5% crystal violet for 30 minutes. The cells were then photographed and counted.

### qRT-PCR

Total RNA of PC-3, DU145, LNCaP, 22RV1, RWPE-1 or BPH1 cells after various treatments was extracted with TRIzol-reagent (Tel-Test; Austin, Texas, USA). First-strand cDNA was synthesized by reverse transcription of 2 μg total RNA using a Takara RNA PCR Kit (TaKaRa, Japan). The primers used were: glyceraldehyde 3-phosphase dehydrogenase (GAPDH): Hs02758991; HIF-1α: Hs00153153; HK2: Hs00606086; PDK1: Hs00176853; PKM: Hs00761782; and LDH-A: Hs01378790 (Invitrogen). The expression levels of target genes were normalized to that of the housekeeping gene GAPDH.

### Statistical analyses

Data from all replicates were presented as the mean ± standard deviation (SD). Comparisons among various groups were performed by two-way analysis of variance (ANOVA), followed by post-hoc pairwise repetitive comparisons using Tukey’s test with Origin 9.0 Lab data analysis and graphing software. Statistical significance was considered as p < 0.05.

### Availability of data and materials

The datasets analyzed during the current study are available from the corresponding author on reasonable request.

## Electronic supplementary material

Data supplements
