# Supplementary material for: Knockdown of HIF-1α by siRNA-expressing plasmid delivered by attenuated Salmonella enhances the antitumor effects of cisplatin on prostate cancer
Source: Sci Rep. 2017 Aug 8;7:7546. doi: 10.1038/s41598-017-07973-4 (PMC5548753; doi:10.1038/s41598-017-07973-4)
Supplement: Supplementary file 1 — Data supplements [file 41598_2017_7973_MOESM1_ESM.pdf]

**Knockdown of HIF-α by siRNA-expressing plasmid delivered by attenuated *Salmonella* enhances the antitumor effects of cisplatin on prostate cancer**

Junlian Gu, Yang Li, Jun Zeng, Bo Wang, Kun Ji, Yufeng Tang, and Qing Sun

**Contents**

**1. Supplemental Methods and Materials**
**2. Supplemental Figures**

## 1. Supplemental Methods and Materials

*Construction of vector expressing hypoxia-inducible factor-1 alpha siRNA*

According to the method used in our previous publications (Ji et al., 2011; Zhang et al., 2007), an siRNA with the sequence 5'-CUGAUGACCAGCAACUUGA-3' (Genbank accession number NM_001530.3) was selected to specifically target hypoxia-inducible factor-1 alpha (HIF-1α) mRNA. The oligonucleotide contains a sense strand of 19 nucleotides followed by a short spacer (loop sequence 5-TTCAAGAGA-3), an antisense strand, and a terminator containing five Ts. Double-stranded DNA oligonucleotides were cloned into pGCsilencerU6/Neo/GFP (Jikai Chemical, Inc.) to generate the si-HIF-1α plasmid (Supplementary Figure 1).

*Plasmids and transfection*

PC-3 cells were plated at a density to achieve 75–85% confluency and transfected with human LDH-A (pCMV6-XL4) cDNA (Origene, Rockville, MD). Additionally, cells were transfected with an empty vector (pcDNA) as a control. HIF-1α-siRNA transfection was performed with Lipofectamine 2000 reagent (Invitrogen, Carlsbad, CA). The transfection protocol used was described previously (Gu et al., 2014; Ji et al., 2011).

*Immunohistochemistry*

The paraffin-embedded cancer tissue sections (4 μm) were deparaffinized, rehydrated, and incubated in 3% $H_2O_2$ to quench an endogenous peroxidase effect. Next, tissue sections were incubated with a serum-blocking solution for 30 min, then incubated with primary antibodies against HIF-1α (Novus) and PCNA (Calbiochem) overnight, washed with phosphate-buffered saline (PBS), and incubated with biotinylated secondary antibody for 60 min. Finally, nuclei were counterstained with hematoxylin and images were captured on a light microscope. Positive and negative cells were counted in 10 random high-power fields for each sample.

*Glucose-uptake and lactate assays*

Glucose uptake was assayed using a Glucose Uptake Assay Kit (Abcam, Cambridge, MA). Briefly, PC-3 cells (1.0 x $10^4$ cells/well) were cultured on 96-well plates and treated as indicated. The PC-3 cells were washed twice with PBS and starved in 100 μL of serum-free F-12K medium (ATCC, lot: 60971339) overnight to promote increased glucose uptake, then preincubated with 100 μL Krebs-Ringer-Phosphate-Hepes buffer containing 2% bovine serum albumin for 40 min. The cells were then incubated with 10 μL of 10 mM 2-deoxyglucose (2-DG) for 20 min. The uptake reaction was performed at 37°C for 1 h and terminated by adding 80 μL extraction buffer, following the manufacturer's instructions. The amount of 2-DG in the test samples, which was proportional to the accumulated 2-DG-6-phosphate (2-DG6P), was calculated using the plotted 2-DG6P standard curve.

Lactate levels in the culture medium of PC-3 cells were measured using the EnzymChrom L-Lactate assay kit (BioAssay Systems) according to the manufacturer's instructions. The data were normalized against the total number of cells.

*Flow cytometry (FCM) analysis*

PC-3 xenografts that received the various treatments were ground and filtered through a cell-filter membrane, and resuspended in 100 μL PBS. Next, the cells were incubated with DNA staining solution containing 5 μL propidium iodide (Beckman Coulter, Fullerton, CA) for 30 min at room temperature in the dark. The cell-cycle distribution of the PC-3 cells was evaluated by FCM using an Epics-XL-MCL flow cytometer (Beckman Coulter).

*Luciferase reporter assay*
The HIF-1α reporter gene, constructed in the pGL3 vector, was obtained from Jiran Laboratories (Jiran Bioengineering, Shanghai, China). Cell lysates from PC-3, DU145, LNCaP, 22RV1, REPE-1, or BPH1 cells were collected 48 h after the transfection, and the luciferase assay was conducted with a Dual-Luciferase Reporter Assay Kit following the manufacturer's instructions (Promega).

*Toxicity assessment*
Possible adverse side effects were assessed based on animal body weight, appearance, behavior, appetite, diarrhea, and survival until they were sacrificed. Organs such as the heart, liver, spleen, lung, and kidney were collected and fixed in a 4% paraformaldehyde solution. Paraffin-embedded sections (4 μm) were obtained for general morphological observation by hematoxylin and eosin (H&E) staining in a blinded manner under a microscope. Serum levels of alanine transaminase (ALT) and aspartate transaminase (AST) were detected using an enzyme-linked immunosorbent assay (ELISA) kit from BioVision according to the manufacturer's instructions.

## 2. Supplemental Results

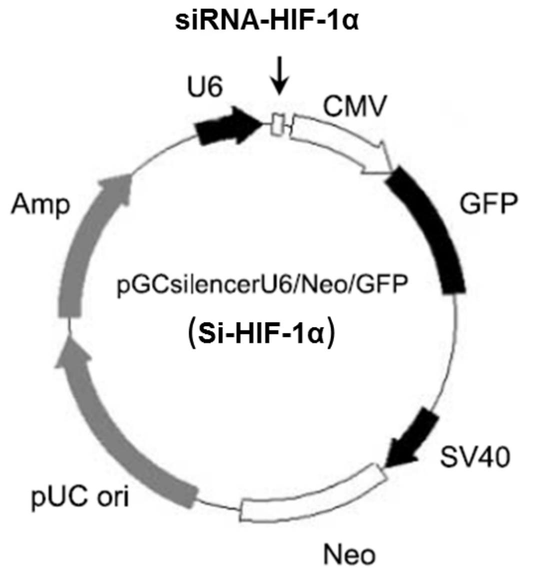


**Supplementary Figure 1. Structure of the si-HIF-1α plasmid containing the HIF-1α small interfering RNA**

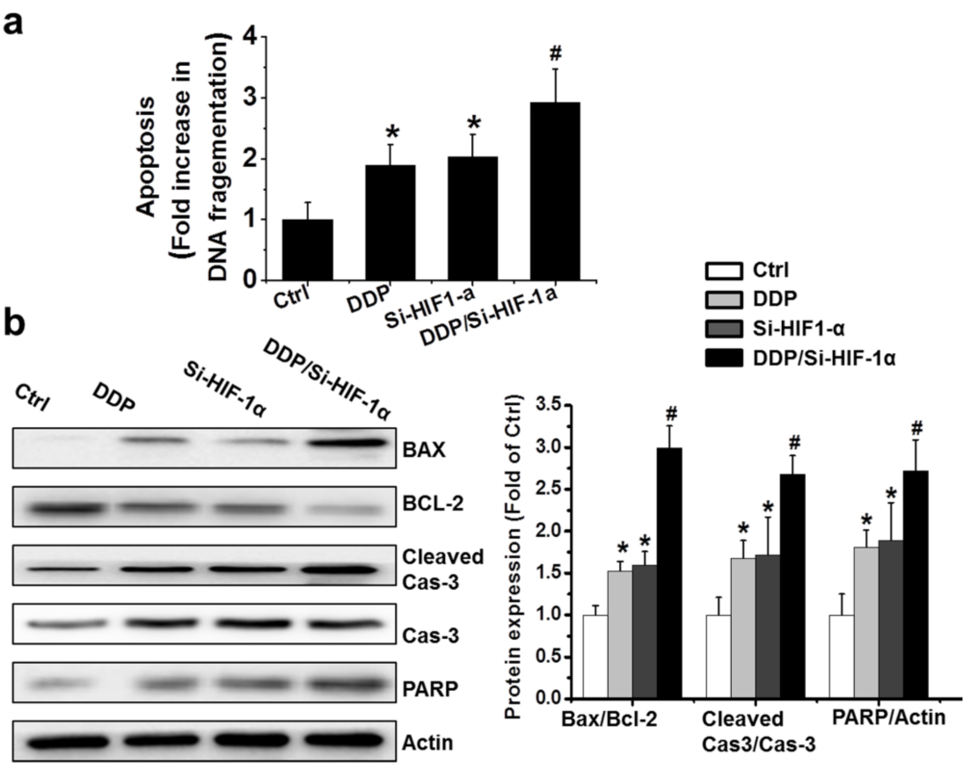


**Supplementary Figure 2. Effects of DDP and/or HIF-1α siRNA treatments on the induction of apoptosis in PC-3 cells**. (a) DNA fragmentation was detected with an apoptosis ELISA assay in PC-3 cells. The cells were transiently transfected with HIF-1α-specific siRNA using Lipofectamine 2000 for 48 h; 24 h after siRNA treatment, the cells were exposed to DDP or vehicle control. After treatment, cell lysates were prepared and used for analysis with the apoptosis ELISA. (b) Protein expression of Bax/Bcl-2 ratio, cleaved caspase-3, and cleaved PARP was examined in PC-3 cells subjected to various treatments, using western blot analysis. Data shown are mean ± SD of three separate experiments. * $p < 0.05$ versus control group; # $p < 0.05$ versus the si-HIF-1α group or DDP group. The original blots are presented in Supplementary Figure 10.

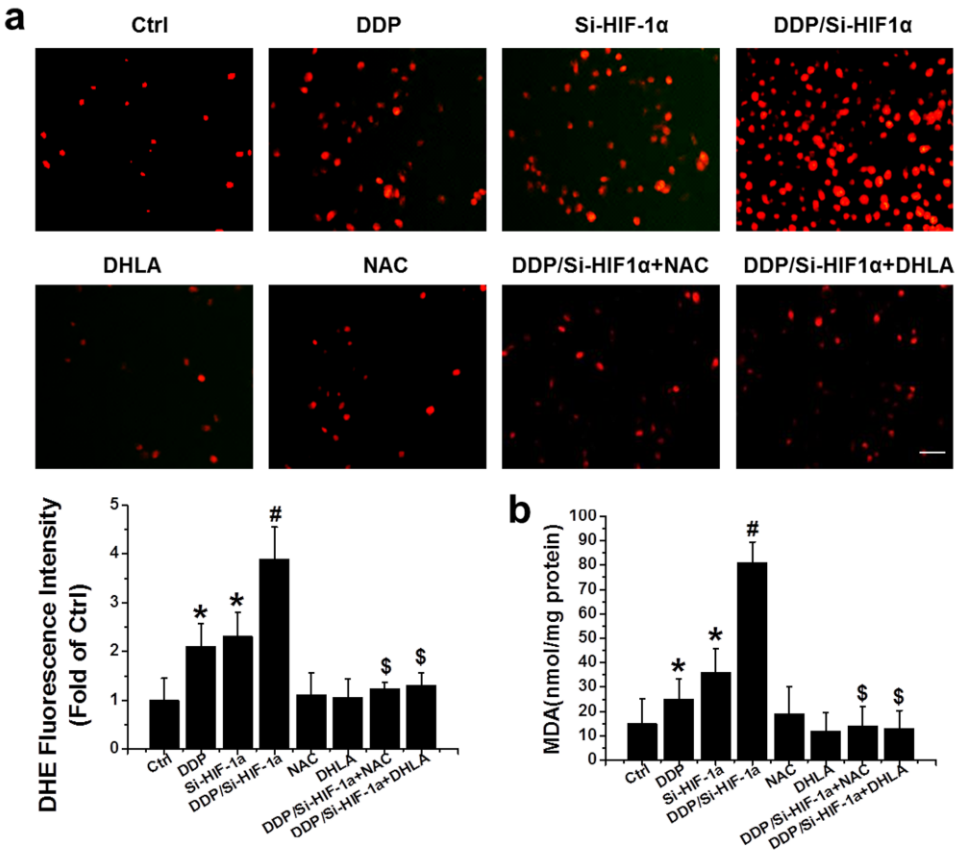


**Supplementary Figure 3. DDP and/or HIF-1α siRNA treatment induced apoptosis of PC-3 cells through ROS overproduction.** (a) PC-3 cells were treated with DDP, si-HIF-1α plasmid, or both, in the presence or absence of NAC (5 mM) or DHLA (0.25 mM) for 24 h. The ROS level of PC-3 cells was monitored by DHE staining (red) and observed under a fluorescence microscope (scale bars, 50 μM). (b) MDA formation of PC-3 cells was examined after different treatments. Data shown are mean ± SD of three separate experiments. * $p < 0.05$ versus control group; # $p < 0.05$ versus si-HIF-1α or DDP group; $ $p < 0.05$ versus DDP/si-HIF-1α group.

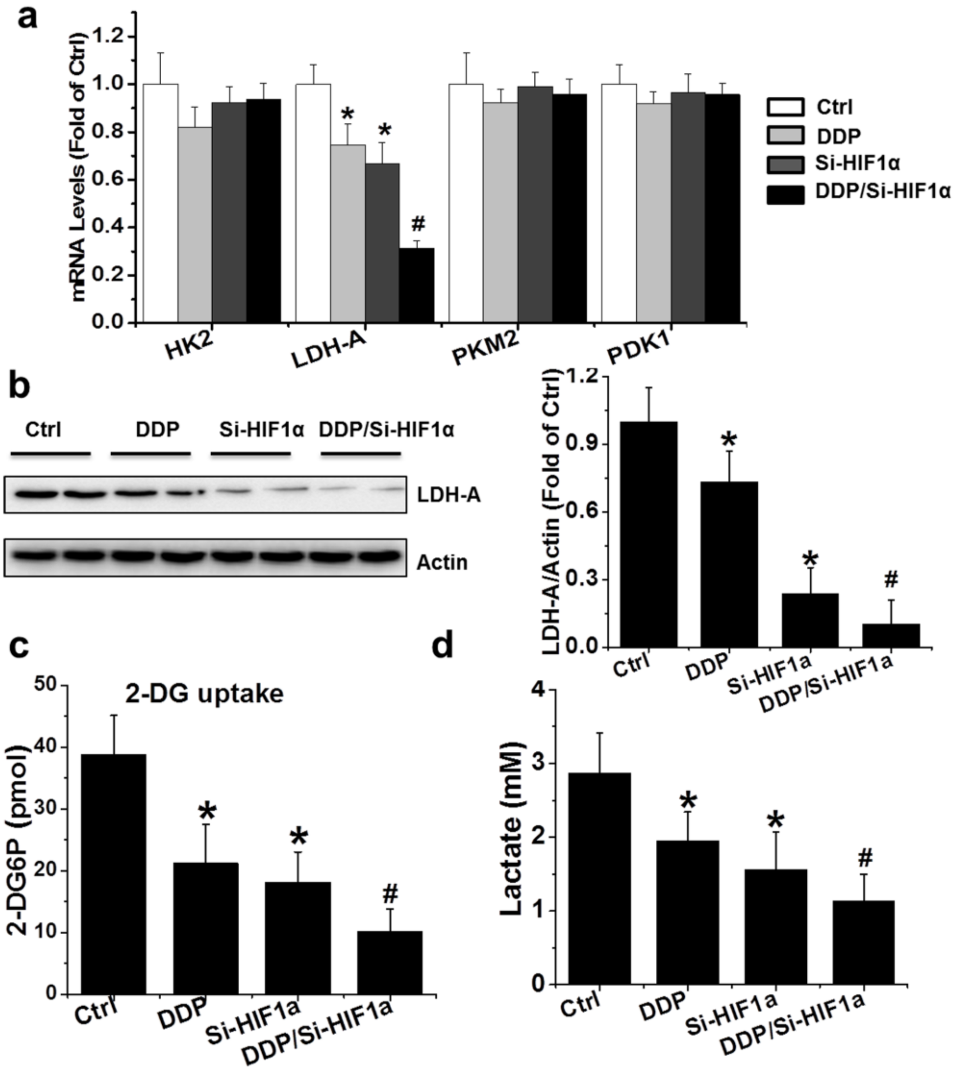


**Supplementary Figure 4. DDP and/or HIF-1α siRNA treatment reduced glycolytic enzyme LDH-A expression, glucose uptake, and lactate accumulation.** (a–b) mRNA and protein expression of HK2, LDH-A, PKM2, and PDK1 was examined with qRT-PCR (a) and western blot assays (b) in PC-3 cells. (c) Cellular glucose uptake was determined by measuring intracellular 2-DG6P after various treatments in PC-3 cells. (d) Lactate accumulation in the media of PC-3 cells after various treatments. Data shown as mean ± SD of three separate experiments. *p < 0.05 versus control group; #p < 0.05 versus si-HIF-1α group or DDP group. The original blots are presented in Supplementary Fig. 10.

S.Fig.5

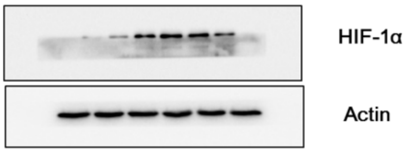


The above orignal gel/blot correspond to those shown in the cropped images presented as Fig. 1A

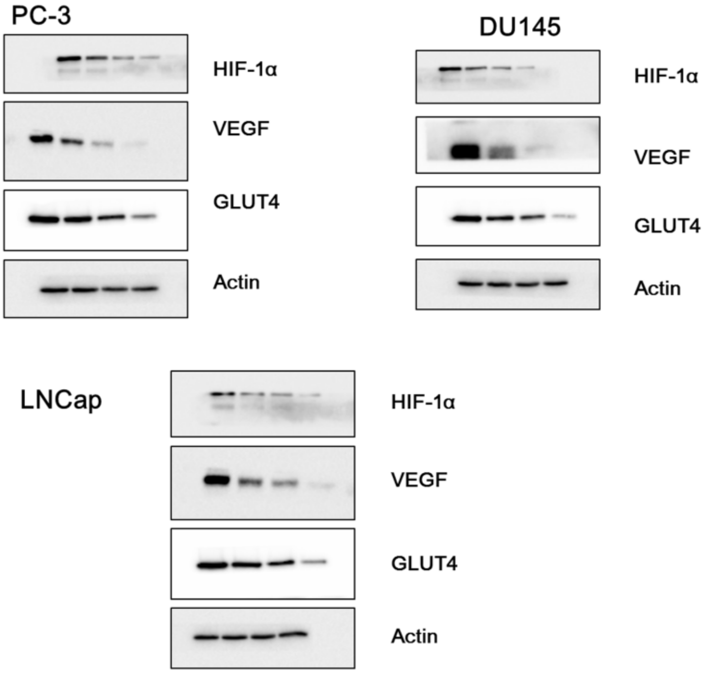


The above original gel/blot correspond to those shown in the cropped images presented as Fig. 1G

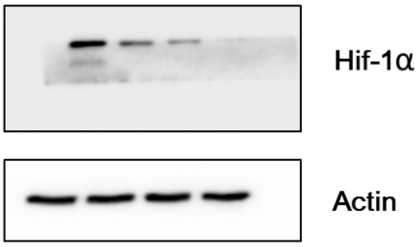


The above orignal gel/blot correspond to those shown in the cropped images presented as Fig. 3D

S.Fig. 7

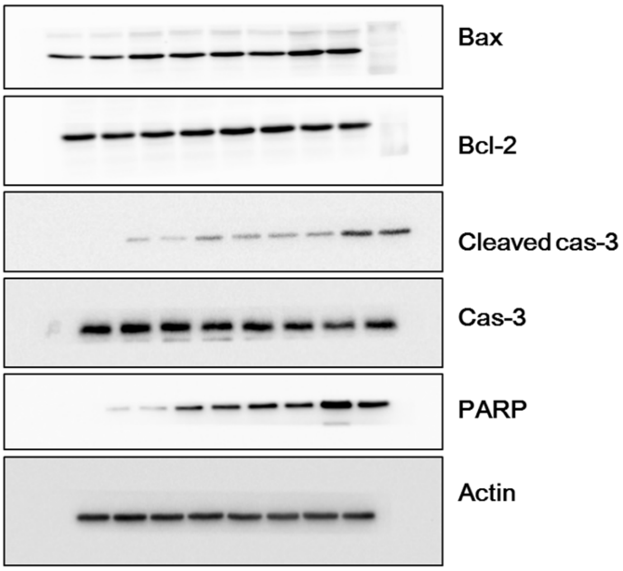


The above original gel/blot correspond to those shown in the cropped images presented as Fig. 4D,E

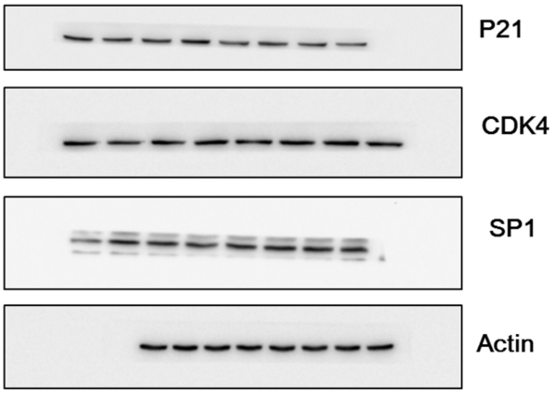


The above original gel/blot correspond to those shown in the cropped images presented as Fig. 4G

S.Fig. 8

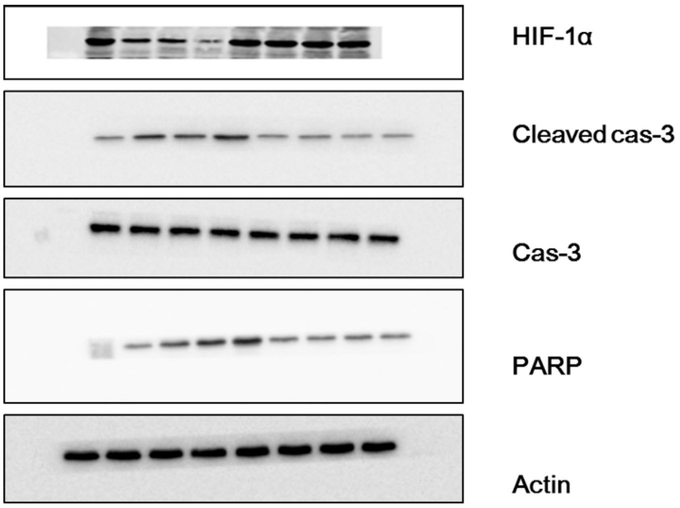


The above original gel/blot correspond to those shown in the cropped images presented as Fig. 5E

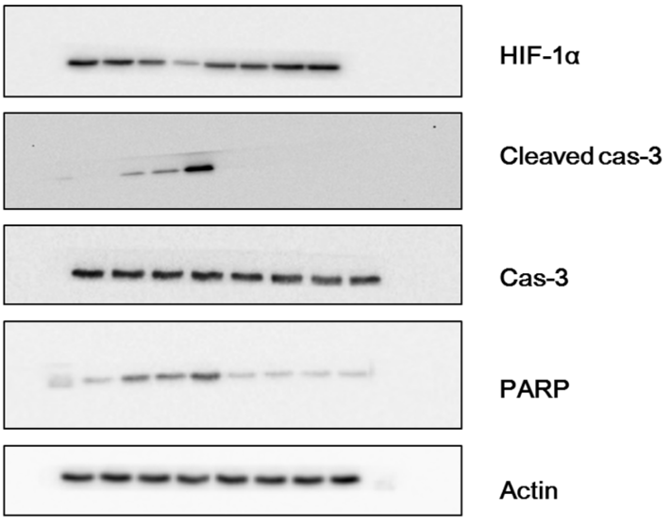


The above original gel/blot correspond to those shown in the cropped images presented as Fig. 5F

S.Fig. 9

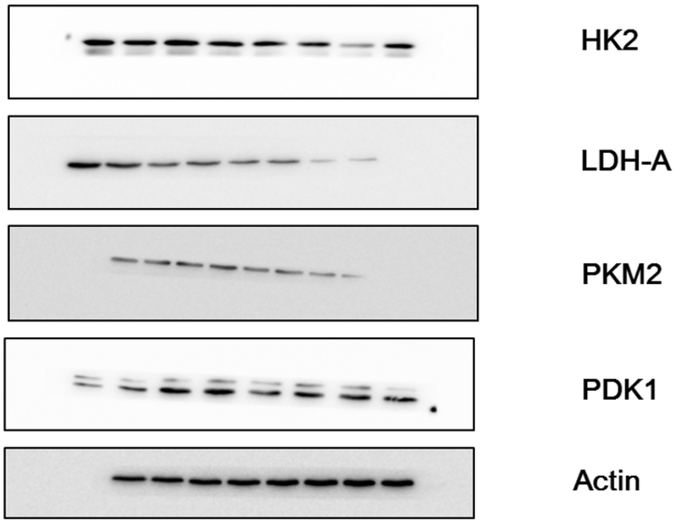


The above original gel/blot correspond to those shown in the cropped images presented as Fig. 6B

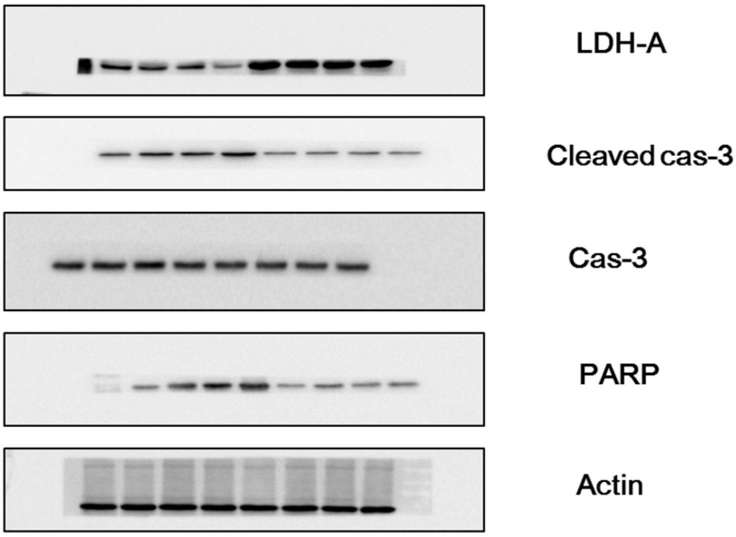


The above original gel/blot correspond to those shown in the cropped images presented as Fig. 6C

S.Fig. 10

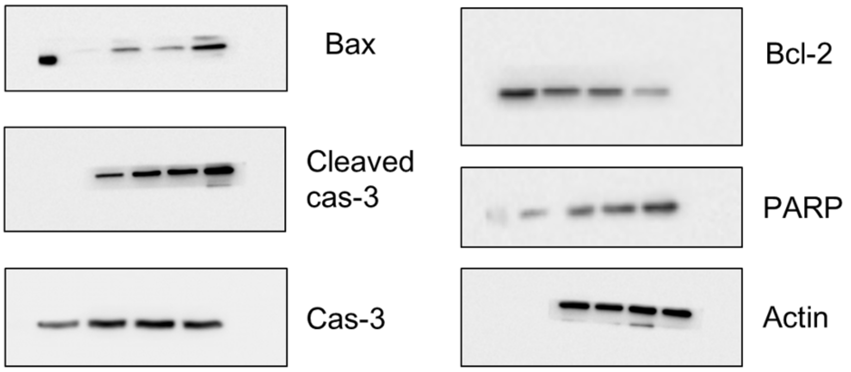


The above original gel/blot correspond to those shown in the cropped images presented as S. Fig. 2

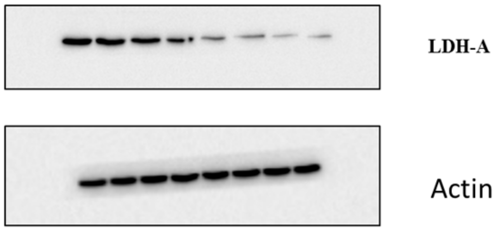


The above original gel/blot correspond to those shown in the cropped images presented as S. Fig. 4
